# Demographic Factors Influencing Eligibility for EMA in South Australia

**DOI:** 10.1111/ajo.70069

**Published:** 2025-11-17

**Authors:** Laura Slade, Jennie Louise, Katina D'Onise, Jodie Dodd

**Affiliations:** ^1^ Women's and Children's Hospital North Adelaide South Australia Australia; ^2^ Robinson Research Institute University of Adelaide Adelaide South Australia Australia; ^3^ Women's and Children's Hospital Research Institute Women's and Children's Hospital North Adelaide South Australia Australia; ^4^ South Australian Health and Medical Research Institute Adelaide South Australia Australia; ^5^ School of Public Health University of Adelaide Adelaide South Australia Australia

**Keywords:** abortion access, healthcare equity, termination of pregnancy

## Abstract

**Background:**

Early medical abortion (EMA) can be performed by administration of mifepristone and misoprostol in an outpatient setting prior to 63 days gestation in Australia. While this is a flexible, efficacious and safe option for abortion, it requires early identification of pregnancy and efficient access to a clinical service. Outpatient EMA with mifepristone and misoprostol was introduced in Australia in 2012 for gestations < 49 days, and extended to 63 days in 2015.

**Methods:**

The laws governing abortion in South Australia mandate routine data collection. A retrospective cohort study was conducted of all registered abortions in South Australia from 2012 to 2020. Women undergoing abortion before 7 weeks gestation between 2012 and 2014 and then from 2015 those undergoing abortion before 9 weeks gestation were considered EMA eligible. Demographic characteristics were then compared using multiple logistic regression.

**Results:**

Women who were eligible for EMA were significantly different from those who were ineligible based on gestational age. Women who were ineligible were more likely to be teenagers, live in rural and remote areas and live in areas of socio‐economic disadvantage. In multivariable logistic regression teenagers were disproportionately less likely to be EMA eligible, with rural women and socially disadvantaged teenagers having the lowest rates of and eligibility for EMA.

**Conclusion:**

Eligibility for EMA was affected by age, rurality and socio‐economic disadvantage. Interventions to improve access should investigate and address the specific barriers facing these groups of women.

## 1 Introduction

Abortion can be affected by medical or surgical methods depending on gestation, indications and the individual woman's preference. Early medical abortion (EMA) is provided in Australia by the use of mifepristone, a progesterone antagonist, and misoprostol, a prostaglandin analogue. Although mifepristone and misoprostol have been used effectively worldwide for many years, introduction into Australia required prolonged and persistent campaigning [1]. In 2013, the combination of mifepristone and misoprostol became available, marketed in Australia as “MS2‐Step.” Initially this was licenced for use to 49 days gestation, extending to 63 days gestation in 2015 [2].

While EMA with mifepristone and misoprostol is a safe and effective method of abortion [3, 4, 5], access is hampered by geographical, financial and legal barriers. Recent estimates from the Pharmaceutical Benefit Scheme (PBS) data demonstrate that 30% of Australian women of reproductive age live in an area without a doctor who is registered to provide EMA [6]. Financial costs, including travel, procedural and other fees not covered by Medicare, and the costs of accommodation away from home are particular barriers for women living in rural and remote areas seeking to access abortion [7, 8].

Laws governing abortion in Australia are slowly removing restrictions to access across jurisdictions. South Australia was the last Australian state to decriminalise abortion in 2021. This removed the long‐standing requirement for abortion to be undertaken in an approved hospital service [9]. Prior to August 2023, MS2‐Step could not be prescribed by Nurse Practitioners or Endorsed Midwives or doctors working in community settings. Registration requirements for practitioners and pharmacies have also been removed, which had previously limited the number of sites able to dispense these medications.

Optimising access to EMA benefits both the individual woman and the healthcare system. Earlier gestation at EMA is associated with higher success rates and lower rates of complications [3, 4]. When compared with surgical termination of pregnancy (STOP), EMA is cheaper than a STOP which involves health service admission, anaesthetic administration and specific equipment. Understanding the factors associated with presentation for abortion at gestations above those eligible for EMA is key to maximising the potential use of this service across Australia and in many similar high‐income countries across the world.

## 2 Methods

In South Australia, abortion was legislated in 1969, with the requirement of mandatory reporting to a centralised register, providing a unique state‐wide dataset across more than 50 years. A retrospective cohort study of all abortions in South Australia was analysed to investigate the impact of demographic factors on gestation and eligibility for EMA. This was defined as < 49 days gestation from 2012 to 2015, and then < 63 days gestation from 2015 to 2020 to align with the regulations surrounding mifepristone prescription. Gestation was recorded as the number of completed weeks. Records with gestations < 3 weeks, or more than 40 weeks were excluded as these were not possible to verify. Abortions for fetal anomalies, and any cases with missing gestation were excluded from this analysis.

Rurality was coded according to the 2016 Australian Statistical Geographical Classification Remoteness Area (AGSC‐RA) system using postcode of residence. Postcodes were also coded according to the Index of Relative Socio‐economic Advantage and Disadvantage (IRASD) (Australian Bureau of Statistics 2018). For regression analyses, this was divided into three categories, with the lowest category being the most disadvantaged and the highest being the least disadvantaged. A teenager is defined as a person aged < 20 years at the time of abortion.

Demographic variables were compared using the chi‐squared test for categorical variables and the *t*‐test for continuous variables. Multivariable logistic regression was used to investigate the effect of changes in demographic variables over time on the proportion of women undergoing abortion who were EMA eligible. Then, to investigate those most and least likely to undergo abortion at EMA eligible gestations, multiple logistic regression was used to estimate the proportion of women undergoing abortion at an EMA eligible gestation according to the varying combinations of demographics. For this analysis IRSAD was coded into terciles, remoteness was grouped to define those in remote and very remote areas, and teenager was again defined as those aged < 20 years.

Ethics approval was obtained from the Department of Health, South Australia, which is the data custodian for the registry, with reference number 2022/HRE00176.

## 3 Results

Women presenting for abortion who were eligible for EMA based on gestation were older and less likely to be teenagers (8.23% vs. 13.77%). A higher proportion of women who were eligible for EMA lived in a major city (73.16% vs. 58.61%). Using the Index of Relative Socio‐Economic Advantage and Disadvantage, fewer women were from the most disadvantaged quintile (27.82% vs. 34.74%) and more were from the most advantaged and least disadvantaged quintiles (11.00% vs. 8.91%) (Table 1).

**TABLE 1:** Demographics by gestation at abortion (*n* (%) unless otherwise specified).

	EMA eligible	EMA ineligible
Total	22046 (56.04%)	17292 (43.96%)
Age (years) (median (IQR))	27 (23, 33)	26 (22, 32)
Teenage	1815 (8.23%)	22381 (13.77%)
Nulliparous	2255 (10.23%)	3601 (20.82%)
Missing parity	8352 (37.88%)	4578 (26.47%)
Remoteness		
Major city	16128 (73.16%)	11864 (58.61%)
Inner regional	3959 (17.96%)	3390 (19.60%)
Outer regional	1270 (5.76%)	1289 (7.45%)
Remote	584 (2.65%)	580 (3.35%)
Very remote	79 (0.36%)	116 (0.67%)
Missing	26 (0.12%)	53 (0.31%)
Index of relative socio‐economic advantage and disadvantage^a^		
Quintile 1	6133 (27.82%)	6008 (34.74%)
Quintile 2	3314 (15.03%)	2797 (16.18%)
Quintile 3	5497 (24.93%)	3774 (21.83%)
Quintile 4	4621 (20.96%)	3108 (17.97%)
Quintile 5	2425 (11.00%)	1541 (8.91%)
Missing	56 (0.25%)	64 (0.37%)
Hospital where abortion performed		
Metropolitan public	7077 (32.10%)	6179 (35.73%)
Metropolitan private	12 (0.05%)	339 (1.96%)
Rural	777 (3.52%)	12 (0.07%)
Missing	14180 (64.32%)	10762 (62.24%)

Abbreviation: EMA, early medical abortion.

^a^ Quintiles—Q1 least advantaged, most disadvantaged, Q5 most advantaged, least disadvantaged.

An increasing proportion of women undergoing abortion were eligible for EMA over time increasing from 29.70% in 2012 to 78.87% in 2020 (Table 2). The proportion of teenage people decreased from 20.51% to 11.93%. The proportion of women from each IRSAD quintile and area of remoteness was stable over time. A higher proportion of women were from quintile 1 representing those with the most disadvantage and least advantage, and most were from a major city (Table 2).

**TABLE 2:** Changes in demographics over time for those presenting at EMA eligible gestation (*n* (%) unless otherwise specified).

	2012	2013	2014	2015	2016	2017	2018	2019	2020
Total	1363 (29.70%)	1201 (26.54%)	1165 (26.04%)	3191 (74.70%)	2935 (70.05%)	2814 (67.51%)	2867 (67.51%)	2932 (67.73%)	3568 (78.87%)
Teenage	955 (20.51%)	1730 (25.13%)	2384 (23.65%)	2420 (20.80%)	2562 (18.48%)	3341 (19.33%)	2982 (19.44%)	2417 (16.78%)	1932 (11.93%)
Index of relative socio‐economic advantage and disadvantage									
Q1^a^	371 (27.22%)	336 (27.98%)	314 (26.95%)	922 (28.89%)	877 (29.88%)	724 (25.73%)	779 (27.17%)	786 (26.81%)	1024 (28.70%)
Q2	177 (12.99%)	168 (13.99%)	146 (12.53%)	474 (14.85%)	457 (15.57%)	443 (15.74%)	439 (15.31%)	468 (15.96%)	542 (15.19%)
Q3	334 (24.50%)	2228 (20.80%)	307 (26.35%)	790 (24.76%)	693 (23.61%)	730 (25.94%)	709 (24.73%)	751 (25.61)	892 (25.00%)
Q4	304 (22.30%)	2228 (20.80%)	251 (21.55%)	667 (20.90%)	572 (19.49%)	606 (21.54%)	615 (21.45%)	623 (21.25%)	732 (20.52%)
Q5	175 (12.84%)	2228 (20.80%)	144 (12.36%)	334 (11.38%)	334 (11.38%)	308 (10.95%)	324 (11.30%)	283 (9.65%)	370 (10.37%)
Missing	2 (0.15%)	2 (0.17%)	3 (0.26%)	4 (0.13%)	2 (0.07%)	3 (0.11%)	11 (0.38%)	21 (0.72%)	8 (0.22%)
Remoteness									
Major city	1040 (76.30%)	902 (75.10%)	879 (75.45%)	2327 (72.92%)	2104 (71.69%)	2042 (72.57%)	2085 (72.72%)	2127 (72.54%)	2622 (73.49%)
Inner regional	212 (15.55%)	211 (17.57%)	189 (16.22%)	565 (17.71%)	533 (18.84%)	534 (18.98%)	537 (18.73%)	540 (18.42%)	618 (17.32%)
Outer regional	67 (4.92%)	49 (4.08%)	58 (4.98%)	204 (6.39%)	182 (6.20%)	154 (5.47%)	166 (5.79%)	170 (5.80%)	220 (6.17%)
Remote	37 (2.71%)	34 (2.83%)	32 (2.75%)	81 (2.54%)	78 (2.66%)	72 (2.56%)	79 (2.76%)	81 (2.76%)	90 (2.52%)
Very remote	5 (0.37%)	3 (0.25%)	4 (0.34%)	10 (0.31%)	15 (0.51%)	9 (0.32%)	8 (0.28%)	12 (0.41%)	13 (0.36%)
Missing	2 (0.15%)	2 (0.17%)	3 (0.26%)	4 (0.13%)	3 (0.10%)	3 (0.11%)	2 (0.07%)	2 (0.07%)	5 (0.14%)

Abbreviation: EMA, early medical abortion.

^a^ Quintiles—Q1 least advantaged, most disadvantaged; Q5 most advantaged, least disadvantaged.

Using univariable logistic regression, teenagers were significantly less likely to undergo abortion at EMA eligible gestations (OR 0.56 [0.53, 0.60]) compared with women aged 20 and above. Using IRSAD (with Quintile 5 representing the most advantaged and least disadvantaged) as the reference, quintile 1 (OR 0.65 [0.60, 0.70]) and quintile 2 (OR 0.75 [0.69, 0.82]) were associated with reduced odds of undergoing abortion at EMA eligible gestations, whereas quintile 3 (0.93 [0.83, 1.00]) and 4 (OR 0.94 [0.87, 1.02]) were associated with no difference in odds (Table 3).

**TABLE 3:** Association of demographic factors with undergoing abortion at gestation eligible for EMA.

Factor	Odds ratio	*p*
Age (continuous)		
Teenage	0.56 [0.53, 0.60]	< 0.001
Index of relative socio‐economic advantage and disadvantage		
Q1	0.65 [0.60, 0.70]	
Q2	0.75 [0.69, 0.82]	
Q3	0.93 [0.86, 1.00]	
Q4	0.94 [0.87, 1.02]	
Q5	Ref	0.0053
Parity	2.00 [1.89, 2.13]	< 0.001
Remoteness		
Major city	Ref	
Inner regional	0.86 [0.82, 0.90]	
Outer regional	0.72 [0.67, 0.79]	
Remote	0.74 [0.66, 0.83]	
Very remote	0.50 [0.38, 0.67]	< 0.001

Abbreviation: EMA, early medical abortion.

**FIGURE 1:**
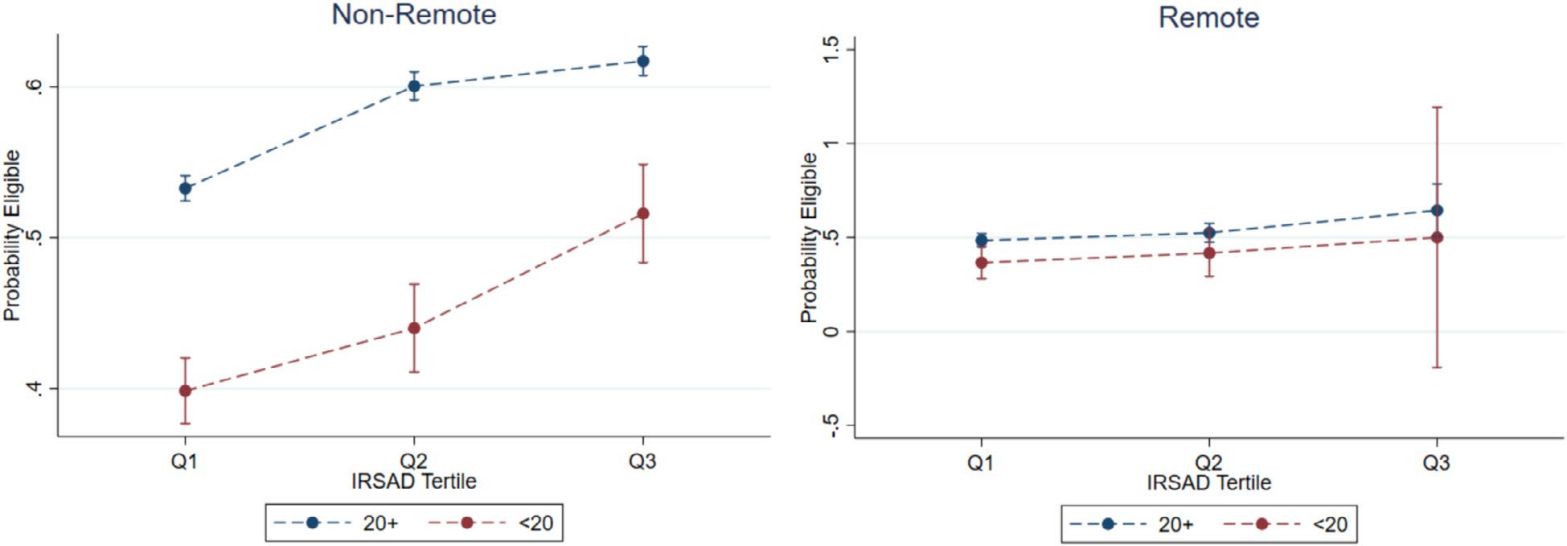
The effect of remoteness, IRSAD and teenage status on the proportion of women presenting at EMA eligible gestations. EMA, early medical abortion; IRSAD, index of relative socio‐economic advantage and disadvantage.

Multivariable logistic regression was used to estimate the proportion of women undergoing abortion at EMA eligible gestations using a combination of demographic variables (Table 4). Individuals least likely to undergo abortion at EMA eligible gestations were teenagers, especially those in the lower IRSAD tercile and those in remote areas. The estimated proportion of those eligible was also lower for remote women and those in the lowest IRSAD tercile for those aged 20 or more (Figure 1).

**TABLE 4:** Estimated proportion of women undergoing abortion at EMA eligible gestations by IRSAD tercile, remoteness and teenage status.

IRSAD tercile	Remote	Teenager	Estimated proportion EMA eligible (%) [95% CI]
1	N	N	53.3% [52.4%, 54.1%]
1	N	Y	39.8% [37.7%, 42.0%]
2	N	N	60.1% [59.1%, 61.0%]
2	N	Y	44.1% [41.1%, 46.9%]
3	N	N	61.7% [60.7%, 62.7%]
3	N	Y	51.6% [48.3%, 54.9%]
1	Y	N	48.4% [44.8%, 52.0%]
1	Y	Y	36.6% [28.1%, 45.1%]
2	Y	N	52.5% [47.5%, 57.4%]
2	Y	Y	41.7% [29.2%, 54.1%]
3	Y	N	64.4% [50.5%, 78.4%]
3	Y	Y	50% [−19.3%, 119.3%]

*Note:* N – no, Y – yes, red – < 50%, yellow – 50%–59%, green – > 60%.

Abbreviations: EMA, early medical abortion; IRSAD, index of relative socio‐economic advantage and disadvantage.

## 4 Discussion

Presentation for abortion at a gestation eligible for EMA varied significantly according to the demographic characteristics considered. The proportion of women presenting at a gestation eligible for EMA increased over time, reflecting adjustments to the regulations in 2015 in which the gestational limit of eligibility increased. However, teenagers, individuals in remote and very remote areas and individuals in the most disadvantaged and least advantaged areas remained less likely to be eligible to undergo EMA at the time of presentation.

The recent changes to legislation in South Australia have removed the requirement for abortion to be provided in an approved medical facility. Prior to this change, almost all abortions were provided by publicly funded clinics, with surgical procedures occurring within public hospitals [10]. Under this system, women eligible for Medicare are not charged for the clinic visit, MS2‐Step medications, or any surgical procedure. Routinely, ultrasound assessment of gestational age is performed by the treating clinician on the same day as the clinic visit at no additional cost.

The current changes in legislation allow any medical practitioner including General Practitioners (GPs), Nurse Practitioners or Endorsed Midwives to provide MS2‐Step from a clinic; however the cost of this will vary depending on the provider. Out‐of‐pocket costs for appointments, external ultrasounds, blood tests, and medications may influence affordability and accessibility for many women.

Telehealth services and have been reported to be highly effective and acceptable to women both in Australia and across the world [11, 12, 13]. This may be particularly beneficial to women in remote areas and to younger women [11, 14], minimising travel requirements and costs. Despite this service being theoretically appealing and appropriate for teenagers, data on the uptake of telemedicine abortion services by teenagers in other countries are conflicting [15, 16]. For those in rural areas, there are other barriers for providers including a lack of training, support or backup and stigma that are not overcome by telehealth services alone [17]. Access to telehealth alone does not negate multiple other logistical, financial and social barriers, disproportionately faced by teenagers, as well as rural and remote women, and those from areas of socio‐economic disadvantage.

There are many women for whom telehealth may not be appropriate or accessible. Studies of telehealth outside of abortion care have highlighted that characteristics such as ethnicity, education and income that are associated with inequitable access to in‐person services apply to telehealth in the same way [18]. There is little data on women from culturally and linguistically diverse (CALD) backgrounds accessing telehealth services, even less on telemedicine abortion services. A survey of Australian General Practitioners highlighted that service delivery for CALD women seeking EMA through primary care is impacted by a lack of multilingual resources and difficulties in accessing appropriate interpreters [19]. Women who experience domestic violence and reproductive coercion may not have a safe space within their home to access telehealth abortion [19].

Rates of pregnancy overall in teenagers in South Australia have been steadily falling with time, although the proportion that undergoes abortion remains high [20]. Rurality and socio‐economic disadvantage are strongly associated with teenage pregnancy rates across Australia as in many areas of the world [21]. Importantly in this analysis, teenagers remained less likely to be EMA eligible regardless of their rurality or IRSAD status. This highlights that interventions to further reduce the rates of teenage pregnancy and improve their access to abortion need to reach those in all areas. Surveys of use of contraception amongst teenagers consistently report high rates of methods burdened by user error, with low uptake of long‐acting reversible contraception (LARC), with little improvement over time [22, 23].

Amongst all women, long‐acting reversible contraception (LARC) use after EMA is associated with a lower rate of repeat EMA within 2 years [24, 25]. A reliance on telehealth services may also impact the uptake of LARC after abortion [26], especially for women in regional and remote areas where it is more difficult to access an appropriate practitioner. This may also be the case if EMA care is centred in General Practice clinics with few GPs providing intrauterine device insertions [27]. Recently, there has been a funding package announced by the Australian Government to support LARC services and reduce consumer costs which have been a significant barrier to usage [28]. Important improvements may also be seen by extending the scope of practice for nurses in rural and regional Australia, an issue that will be addressed by the ORIENT trial, a pragmatic cluster‐randomised controlled trial of LARC and EMA via nurse‐led models of care in rural and regional Australia [29]. If successful, this will provide evidence of an effective model of care for many women.

### 4.1 Strengths and Weaknesses

This analysis involves one of the largest and most comprehensive population‐based cohorts of abortion data in the world. Few data were excluded because of implausible or missing data. This dataset did not have information on other important variables that predict access and equity in health care including income status, education level and culturally and linguistically diverse status. The specific reasons that abortion was undertaken at a later gestational age in these high‐risk groups are beyond the scope of this analysis.

The coding for remoteness and Index of Relative Socio‐Economic Advantage and Disadvantage was based on 2016 postcode data which may have changed over time. This dataset may also be missing women who travelled interstate to access abortion providers privately. Because the laws in South Australia mandated that abortion had to be carried out inside an approved medical facility [30], private telehealth providers required that women from South Australia had an interstate address where the medications could be received and ingested, limiting the number of women that would have been able to undergo abortion by this method. We were unable to examine other important factors associated with abortion access and availability such as educational qualification, Medicare status, recipient of social benefits or women with economic disadvantage.

## 5 Conclusion

Demographic variables equating socio‐economic advantage were associated with both presentation for abortion at a gestation eligible for EMA and the subsequent choice of EMA if eligible. Overall, teenagers were the least likely population group to be eligible for EMA. Although changes in South Australia's legislation now enable a variety of options for women to access EMA, each is associated with logistical challenges. Disproportionate access to abortion services will not be resolved by removing legal barriers alone.

## Conflicts of Interest

The authors declare no conflicts of interest.

## Data Availability

Data are held by the Department of Health South Australia and are only available with ethics approval upon reasonable request.
